# Synthesis and ethylene polymerization reaction of dendritic titanium catalysts

**DOI:** 10.1080/15685551.2020.1868666

**Published:** 2021-01-18

**Authors:** Tianyu Lan, Liduo Chen, Na Zhang, Jun Wang

**Affiliations:** a College of Chemistry and Chemical Engineering, Northeast Petroleum University, Heilongjiang, China; b Heilongjiang Provincial Key Laboratory of Polymeric Composite Materials,Qiqihar University, China

**Keywords:** Dendrimer, 3, 5-di-tert-butylsalicylaldehyde, catalytic activity, ethylene polymerization, super high molecular weight

## Abstract

The 1.0 G dendrimer (C_22_H_48_N_10_O_4_),3,5-di-tert-butylsalicylaldehyde and TiCl_4_ · 2THF were used as the synthetic materials, and the dendritic salicylaldehyde imide ligand with substituent hindrance and its titanium catalyst were synthesized by the condensation reaction of schiff base. The structure of the synthesized products was characterized by infrared spectroscopy, nuclear magnetic resonance hydrogen spectroscopy, ultraviolet spectroscopy, electrospray mass spectrometry, and inductively coupled plasma mass spectrometry, The actual structure is consistent with the theoretical design structure. Activated methylaluminoxane (MAO) was used as a catalyst precursor for ethylene polymerization in the process of ethylene catalytic. The effects of ethylene polymerization were studied in terms of the Al/Ti molar ratio, reaction time, reaction temperature, polymerization pressure, and ligand structure of the catalyst. The results show at the reaction temperature of 25°C, the reaction time was 30 min, and the ethylene pressure was 1.0 MPa and Al/Ti was 1,000, the catalytic activity can reach 78.56 kg PE/(mol Ti.h). Furthermore, high-temperature GPC-IR, DSC, and torque rheometer were used to characterized the microstructure, thermal properties, and viscoelastic state of polyethylene samples obtained. The results showed that the product was ultra-high-molecular-weight polyethylene.

## 1. Introduction

Ultra-high molecular weight polyethylene (UHMWPE) is a linear structure polyethylene with a molecular weight greater than 1 million. The UHMWPE exhibits advantages of impact resistance, abrasion resistance, chemical resistance, self-lubrication, and cold resistance compared with ordinary polyethylene. The above-mentioned excellent performances make UHMWPE an excellent engineering thermoplastic, and the UHMWPE has been widely and importantly used in many fields [1]. These excellent properties of UHMWPE are closely related to its molecular weight. The catalyst used in the polymerization process is a key factor for obtaining high molecular weight. In the present, the catalysts used to synthesize UHMWPE include Ziegler-Natta catalysts [2–5], metallocene catalysts [6–10], non-metallocene catalysts [11–14], and composite catalysts [15–17]. Composite catalysts have attracted much attention because of the simple synthesis process and high catalytic efficiency. More and more scientists and companies are committed to the synthesis of composite catalysts and the research of the catalytic performance.

The FI complex catalyst [18–20] produced by Mitsui Corporation of Japan is a typical representative of the new composite catalysts. The FI catalyst was designed and developed by the Fujita team in Japan with a ligand-centric concept. It is characterized by simple synthesis, mild reaction conditions, and high ethylene polymerization activity [21]. Ligand structure with rich electronic properties is the key to achieve high catalytic activity [22–24]. UHMWPE can be synthesized by changing the ligand structures. Among the FI catalysts, dendritic macromolecular catalysts [25–28] are relatively special, which can accurately control the number and positions of catalytic active points, so their catalytic performance has been widely studied. In recent years, we have synthesized a series of transition metal catalysts with dendrimers as the framework and used them to catalyze the polymerization of ethylene. It was found that this type of catalyst presents an excellent performance in catalyzing the polymerization of ethylene. At the same time, the end groups of the catalyst and the chain length of the alkyl group at the molecular cavity directly affect the catalytic activity of the catalyst [29–33]. In this paper, a dendritic salicylaldimine titanium catalyst with a large steric hindrance was designed and synthesized. The catalytic conditions for the preparation of UHMWPE were studied, and the influence of the catalyst structure on the catalytic activity of the catalyst and performance of the product was investigated.

## 2. Experimental section

### 2.1 Reagents and instruments

1. 3,5-di-tert-butylsalicylaldehyde, (analytical pure, Aladdin Co., Ltd), tetrahydrofuran, toluene, n-hexane, dichloromethane (analytically pure, Tianjin Comiou Co., Ltd) were used as reagents. Tetrahydrofuran and toluene were dried by refluxing sodium wire/benzophenone under the protection of argon before use. Dichloromethane was used after drying with CAH_2_. MAO (10% toluene solution), Aladdin Co., Ltd), TiCl_4_.2THF (Aladdin), methanol (analytically pure, Tianjin Kemeiou Chemical Reagent Co., Ltd.), and ethylene (polymerization grade, Sinopec Daqing Petrochemical Co., Ltd.) were used after the 4A zeolite drying treatment. 1.0 G dendritic macromolecules were synthesized in the laboratory [34]. All operations were carried out under argon atmosphere by using standard Schlenk technology, where the solvent was steamed.

The equipment used includes Fourier transform infrared spectrometer (Vector 22, Bruker, Switzerland), Micr OTOF-Q II electrospray ionization mass spectrometer (ESI-MS, Bruker, USA), Inov-400 MHz NMR instrument (Varian Corporation, USA), UV-1700pharmaspec type UV-visible spectrophotometer (Shenzhen Comija Instrument And Equipment Co., Ltd.), Agilent 8800 inductively coupled plasma mass spectrometer (Agilent, USA), H-NMR, Bruker AVANCE spectrometer with TMS as internal table standard and scanning frequency of 500 MHz.MS; Elemental analysis, EA-1106 analyzer; Pl-GPC220 high-temperature gel chromatograph (Beijing Pulitech Co., Ltd.); CL800S glove box, Chengdu Delis Industrial Co., Ltd; The melting and crystallization temperatures were determined using the STA 449 F3 Jupiter differential scanning thermal analyzer (DSC). The heating rate was 10 K min under a nitrogen atmosphere. The viscoelasticity of polyethylene was measured using the Thermo Haake Rheostress 600 torque rheometer at 220, a frequency of 1 Hz, and maximum swing stress of 10 kPa.

### 2.2 Synthesis of dendritic salicylaldimine titanium catalyst

#### 2.2.1 Synthesis of ligand

A magnetic stirring bar was placed in a 250 mL three-necked flask in a glove box. The flask was added with anhydrous sodium sulfate (3.0 g) and slowly with 3,5-di-tert-butylsalicylaldehyde (8.8 g, 37.61 mmol). Under a nitrogen atmosphere, the flask was connected to the double-row tube and pumped thrice, and absolute ethanol (20 mL) was injected into it. Then, stirring and heating were carried out. When the temperature reached 78°C, ethanol (50 mL), and 1.0 G PAMAM (2.46 g, 4.76 mmol) were injected for 12 h followed by filtering. The obtained liquid was precipitated using ether as the precipitant, and the yellow solid precipitate was collected and dried in a vacuum at 50°C. A light-yellow solid powder, which is the dendritic salicylaldimine ligand, was obtained with a yield of 62%. ESI-MS (me, Relative Strength, %):1381.9923 (M^+^), M-Elemental analysis C_82_H_128_N_10_O_8_ (1380.99):C, 71.27; H, 9.34; N, 10.14.Elemental analysis:C, 71.22; H, 9.28; N, 11.46;O,10.20.

**Figure 1.**
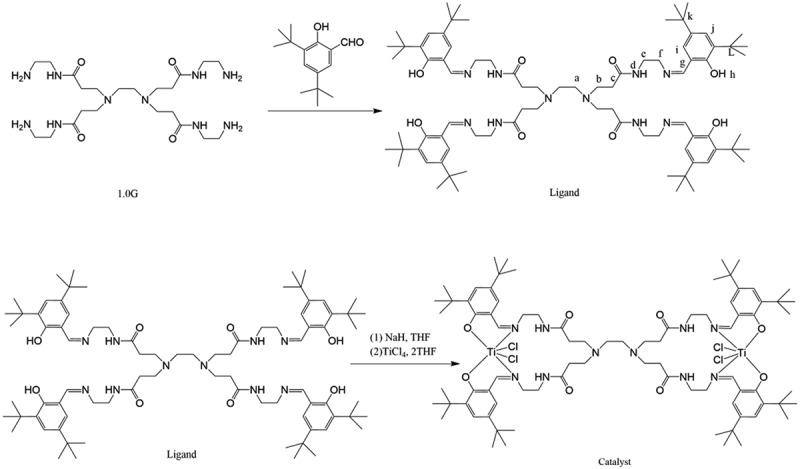
Schematic route for the preparation of dendrimer-supported Ti complex

The synthetic route and the structure of the ligand are shown in Figure 1 as follows:

#### 2.2.2 Synthesis of dendritic titanium catalyst

In a vacuum glove box, ligand (1.3 mmol) was added to THF (100 mL). After full dissolution, NAH (5.2 mmol) was added to the mixture and stirred for 24 h at 25°C. A yellow solid powder was precipitated in the solution. TiCl_4_ · 2THF (2.45 mmol) was added to this solution, and stirring was continued at 25°C for 24 h. Then, the mixture was filtered, and the precipitate was extracted and purified by dichloromethane, washed with toluene, and finally added with n-hexane to precipitate a light-yellow solid powder. Vacuum drying, yield 35%.ESI-MS (me, Relative Strength, %):1616.73 (M^+^). C_82_H_124_Cl_4_N_10_O_8_Ti_2_ (1615.49): Elemental analysis: Ti, 5.93, ICP-AES:Ti 5.90.

The synthesis route and the structure of the titanium catalyst are shown in Figure 1.

#### 2.2.3 Ethylene polymerization

The ethylene polymerization reaction was carried out in a 250 mL stainless steel reactor with magnetic stirring. The reactor was heated under vacuum for 2 h at 160°C and subsequently allowed to cool to the room temperature. The reactor was flushed with ethylene three times. Solvent, the desired amount of cocatalyst, and solution of the metal complex (0.8 μmol/mL, 10 mL) (The total volume was50 mL) was added to the reactor in this order under an ethylene atmosphere, filled with ethylene to the set pressure, and subjected to polymerization reaction at the specified temperature. The reaction was continued for the expect time, the temperature was reduced, the pressure was relieved, and the polymerization reaction was terminated using acidified ethanol with a mass fraction of 10%. Then, the mixture was filtered, and the white solid powder was washed with ethanol. The obtained polyethylene was vacuum dried at 50°C, calculates the catalyst activity of the catalyst. The molecular weight of polyethylene was determined using the viscosity method and gel chromatography (GPC). The former-used decalin as a solvent at 135 ± 0.1°C by using Uzbekistan viscometer, where *η *= 6.77 × 10^−2^(M*η*) ^0.67^ [28]. The viscosity-average molecular weight Mη of polyethylene was calculated. The latter was measured on a PL-220 high-temperature GPC at 140°C with 1,2,4-trichlorobenzene as the mobile phase.

## 3 Results and discussion

### 3.1 FTIR analysis of the ligand and the metal catalyst

**Figure 2.**
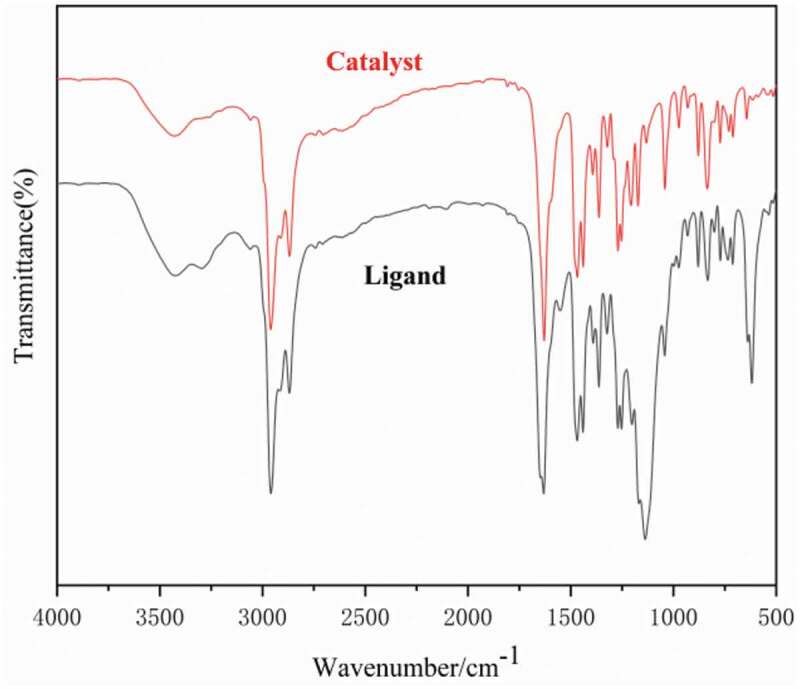
IR spectra of the 3,5-di-tert-butylsalicylaldehyde ligands and titanium complexes

Adopt Vector 22 Fourier Transform of Swiss Bruker Company Infrared spectroscopy was used for the analysis of dendritic 3,5-di-tert-butylsalicylaldehyde ligand and catalyst. As shown in Figure 2, the peak at 3,429 cm-1 can be assigned to the – OH stretching vibrations. The characteristic absorption peak at 2,959 cm-1 can be assigned to the – CH2 – the vibration of the ligand skeleton. A sharp peak was observed near 1,206 cm-1 and can be assigned to the – C–O – vibration. In addition, the absorption peak near 1,467 cm-1 can be assigned to the – C = C – of the benzene ring skeleton in ligand L. The sharp peak observed at 1,633 cm-1 can be assigned to the – C = N – vibration of the dendritic 3,5-di-tert-butylsalicylaldehyde ligand, indicating that the terminal amine group of 1.0 G PAMAM underwent the Schiff base reaction with the aldehyde group of salicylaldehyde to form a dendritic3,5-di-tert-butylsalicylaldehyde ligand [35]. Moreover, the comparison of the infrared spectra of ligand L and complex C shows that after the dendritic 3,5-di-tert-butylsalicylaldehyde ligand is coordinated with metallic titanium, the stretching vibration absorption peak of the catalyst C = N shifted to the low displacement direction and appeared at 1,628 cm-1.

### 3.2 1 H NMR analysis of the ligand and metal catalyst

**Figure 3.**
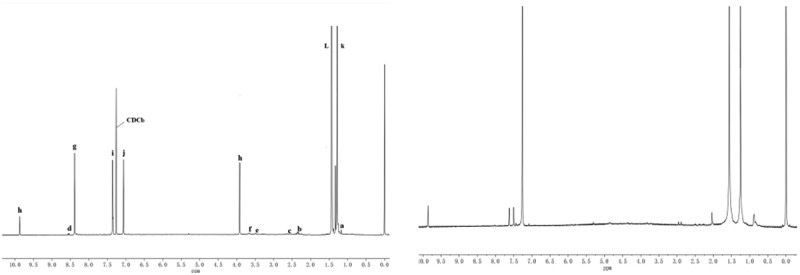
1H-NMR spectra of (a)dendritic 3,5-di-tert-butylsalicylaldehyde ligand and (b) titanium metal catalyst

As shown in Figure 3, the INOV-400 MHz nuclear magnetic vibration instrument was used to characterize the 1 H NMR of the synthesized of Dendritic salicylaldimine ligands and catalysts.

Figure 3(a) shows the 1 H NMR spectrum of the dendritic 3,5-di-tert-butylsal-icylaldehyde ligand, indicating that the characteristic peak of hydrogen proton corresponding to the imine structure g appears at the chemical shift *δ *= 8.25. The characteristic peaks of hydrogen protons corresponding to i and j on the benzene ring appeared at chemical shift *δ *= 7.05–7.62. The chemical shifts *δ *= 1.24, *δ *= 2.63, *δ *= 3.01, *δ *= 3.20, and *δ *= 3.84 correspond to the characteristic peaks of hydrogen protons at a, b, c, e, and f of the L skeleton, respectively. The characteristic peak of hydrogen proton corresponding to d of the amide appeared at chemical shift *δ *= 8.08. The characteristic peak of hydrogen proton corresponding to h at the hydroxyl group of the benzene ring appeared at chemical shift *δ *= 4.02 and *δ *= 9.90, indicating the occurrence of the condensation reaction.

Figure 3(b) shows the 1 H NMR spectrum of the dendritic 3,5-di-tert-buty-lsalicylaldehyde titanium catalyst. Results show that the 1 H NMR spectrum of the catalyst was broadened. After the formation of the complex, the signal peaks of H-a, H-b, H-c, H-d, and H-g shifted slightly to the low field under the influence of metal titanium. The hydrogen proton characteristic peak δ = 4.01 corresponding to the hydroxyl hydrogen proton of benzene ring disappeared. It is indicated that the hydroxyl group lost the hydrogen atom to participate in the coordination. The number of protons did not change, indicating that the reaction proceeded according to the reaction process shown in Figure 1.

### 3.3 MS analysis of synthetic ligands and metal catalysts

**Figure 4.**
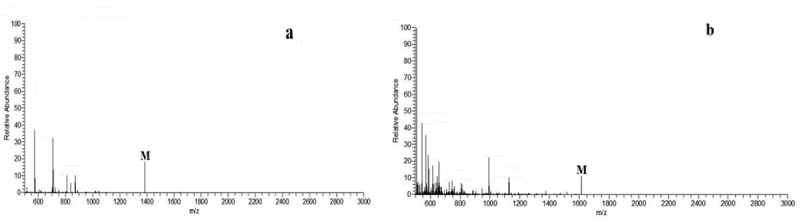
MS spectra of dendritic 3,5-di-tert-butylsalicylaldehyde ligand and its titanium metal catalyst

The mass spectra of the synthesized dendritic 3,5-di-tert-butylsalicylaldehyde

ligand and its titanium metal catalyst were characterized by Bruker’s micr OTOF-Q II electrospray ionization mass spectrometer. The result is shown in Figure 4. The quasi-molecular ion peaks of the dendritic 3,5-dichlorosalicylic aldehyde ligand and its titanium metal catalyst can be observed in the mass spectrum, where the quasi-molecular ion peak of ligand L [M]^+^ appears at m/z = 1381.99. The quasi-molecular ion peak [M]^+^ of catalyst C appeared at m/z = 1616.73。

### 3.4 UV spectra analysis of the ligands and metal catalyst

**Figure 5.**
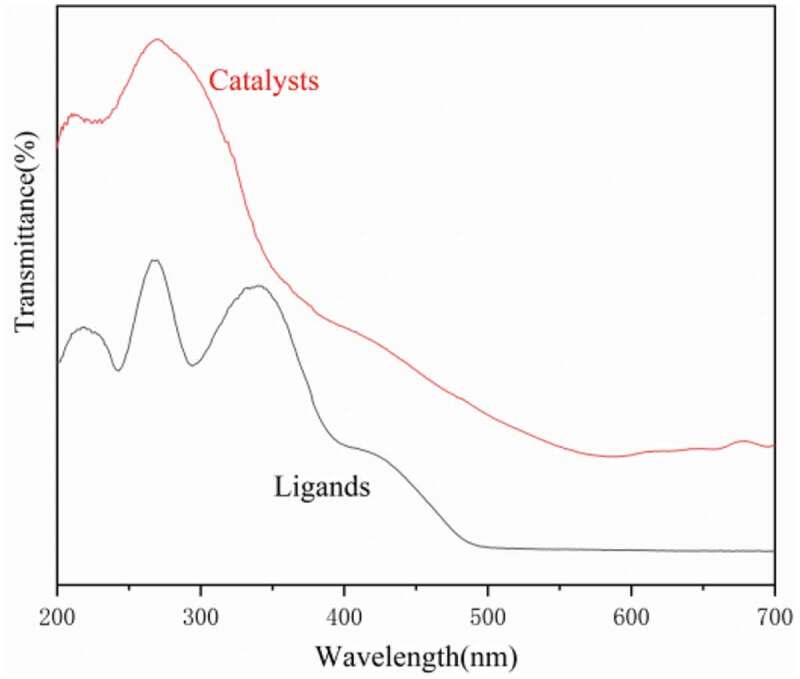
UV spectra of dendritic 3,5-di-tert-butylsalicylaldehyde ligand and titanium catalyst

The UV-visible spectra of the dendritic 3,5-di-tert-butylsalicylaldehyde ligand and its titanium metal catalyst were characterized using the UV-1700 PharmaSpec ultraviolet-visible spectrophotometer. The results are shown in Figure 5. Three absorption bands were observed at approximately 226.5, 272.5, and 350.5 nm in dendritic 3,5-di-tert-butylsalicylaldehyde ligand L. The band at 226.5 nm can be assigned to the π→π* transition of the C = O of the ligand skeleton. The K band at 272.5 nm can be assigned to the conjugation of a benzene ring and C = N. The B band of the benzene ring was masked by the K band. The band around 350.5 nm can be assigned to the π→π* transition R of C = N generated after the reaction. In comparison with the three absorption bands of L near226.5, 272.5, and 350.5 nm, the R band representing the π →π* transition of C = N in the ultraviolet spectrum of the titanium complex C was very weakened and cannot be observed. The K band (272.5 nm) of the benzene ring conjugated with C = N was also blue-shifted. This phenomenon occurred, because the coordination of the titanium atom with the N atom destroyed the conjugated system formed by the C = N bond and the benzene ring. Subsequently, the maximum absorption wavelength decreased, the molar absorption coefficient also decreased, and the band intensity was weakened or even disappeared.

### 3.5 Effect of reaction parameters on the catalyst activity and relative molecular mass

Toluene was used as a solvent, and MAO was used as a co-catalyst in the ethylene polymerization [36]. To probe the effect of reaction parameters on the ethylene polymerization behaviors, we investigated the complexes by changing the reaction temperature, the concentration of MAO, the polymerization time, and ethylene pressure. The detailed results are summarized in Table I.

Reaction conditions: 8 μmol catalyst and 50 mL toluene

Based on entries 4, 8, and 9 in Table 1, as the polymerization temperature increased, the activity of the catalytic system decreased, and the viscosity-average molecular weight gradually decreased. The best catalytic activity was observed at 25°C. This phenomenon occurred possibly because as the reaction temperature rises, the motion rate between molecules of the system will also increase, and the possibility of collision between ethylene monomer and the active site will also increase. Meanwhile, the catalytic activity will also increase. When the reaction temperature reaches a certain critical point, too high a temperature will make ethylene monomer not easy to dissolve in the solvent, so the activity of catalyst will decrease with the increase of the reaction temperature. Hence, both the catalytic activity and molecular weight decreased.

**Table 1.** Results of ethylene polymerization catalyzed by the catalyst

Entry	Ligand	n(AL)/n(Ti)	Time/min		Temperature/°C	Pc2h4/Mpa	Activity/(KgPE.mol^−1^Ti.h^−^[^1]^)	10^−6^M_v_
1	C	0	30	25		1.0	– –	– –
2	C	500	30	25		1.0	10.67	1.41
3	C	800	30	25		1.0	22.46	1.44
4	C	1000	30	25		1.0	78.56	1.48
5	C	1500	30	25		1.0	59.21	1.42
6	C	1000	60	25		1.0	51.23	1.51
7	C	1000	120	25		1.0	36.42	1.54
8	C	1000	30	45		1.0	55.13	1.43
9	C	1000	30	65		1.0	46.86	1.39
10	C	1000	30	25		0.3	26.34	1.31
11	C	1000	30	25		0.5	55.88	1.40

Based on entries 4, 6, and 7 in Table 1, with the increase of polymerization time, the activity of the catalytic system decreased, whereas the viscosity-average molecular weight increased, but the change was not large. This is because, as the polymerization time extends, there are more and more polymers in the system, which may not only embed part of the active center but also affect the diffusion of monomers in the solvent, and reduce the concentration of monomers around the active center, so that the polymerization activity becomes lower and lower. Therefore, the polymerization time is preferably 30 min.

Based on entries 4, 10, and 11 in Table 1, with the increase of ethylene pressure, the activity of the catalytic system increased. This finding occurred because of the concentration of ethylene in the catalytic system, the probability of ethylene colliding with the catalytic active center, and the chain growth rate all increased. Moreover, the chain transfer was effectively inhibited, thus increasing the catalytic activity and viscosity-average molecular weight.

Based on the catalytic antisense of entries 1–11 in Table 1, the Al/Ti molar ratio has a greater influence on ethylene polymerization. Without MAO, the catalytic system had no activity. As the Al/Ti molar ratio increased, the catalytic activity and the viscosity-average molecular weight of polyethylene gradually increased. When n(Al)/n(Ti) was 1,000, the catalytic activity reached the maximum value of 78.56 kg PE/(mol Ti.h). However, when n(Al)/n(Ti) increased to 1,500, both the activity and molecular weight decreased. This finding was recorded possibly because the increase of MAO increased the chain transfer rate, thus decreasing the molecular weight of the polymerized product. Therefore, the maximum n(Al)/n(Ti) should be set to 1,000.

### 3.6 Effect of catalyst structure on the catalyst activity and relative molecular mass

Reaction conditions: 8 lmol catalyst, Al/Ni 1000, toluene 50 mL, 30 min, 25°C,1.0 MPa ethylene.

**Figure 6.**
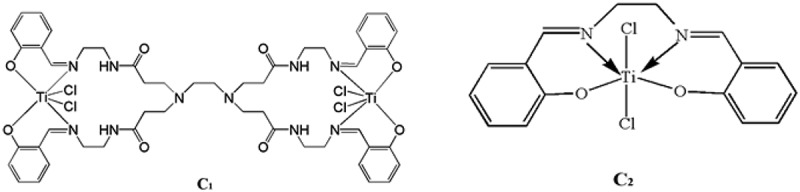
Structure of the metal complexes C_1_ and C_2._

The titanium catalysts C, C_1_ [28], and C_2_ [37]were used as the research objects (Figure 1 and 6) to investigate the effect of the catalyst structure on the performance of ethylene polymerization. Under the optimal reaction conditions, three kinds of salicylaldimine titanium were obtained. The results of ethylene polymerization catalyzed by themic storage and loss modulu catalyst are shown in the Table 2 show that dendritic titanium catalysts C and C_1_ have much higher activity for polymerizing ethylene than non-dendritic titanium catalyst C_2_ because of the dendritic structure in the same molecule. The local concentration of the active sites of the macromolecular catalyst precursor is high. The activity of the dendritic catalyst C with large volume hindrance and the molecular weight of the resulting polyethylene were higher than those of the dendritic catalyst C_1_. This is because the 3,5-di-tert-butylsalicylaldehyde complexes. Among them, the larger steric hindrance can effectively inhibit the β–H elimination reaction during the catalysis of ethylene. The bulky substituents shield the axial plane and prevent chain termination, which is beneficial to the production of polymers, while the steric hindrance decreased. The catalyst easily caused chain termination reaction. The dendritic titanium-based catalyst had a good catalytic activity for ethylene, and the steric hindrance of the substituents can largely control the activity of the catalytic system and the molecular weight of the polymerized product.

### 3.7 Characterization of polyethylene structure

**Figure 7.**
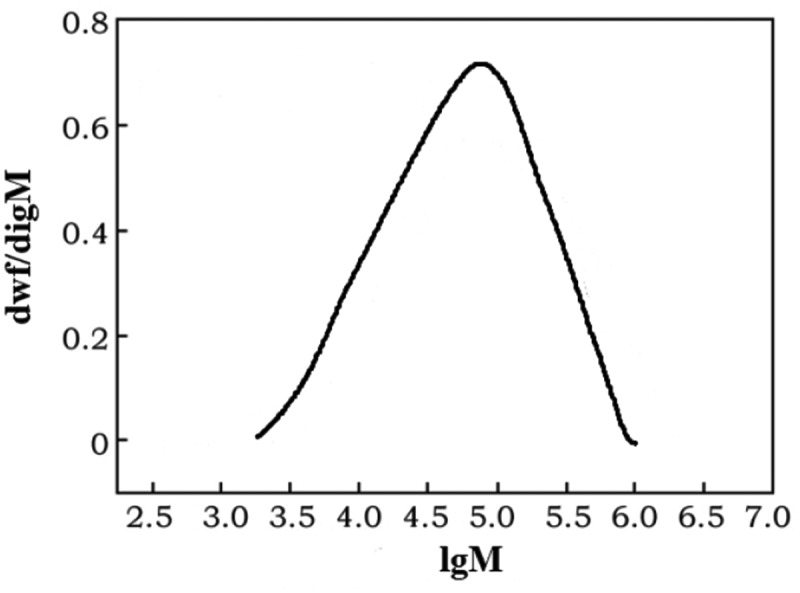
GPC of PE (entry 4 in Table 1)

Figure 7 shows the high-temperature GPC spectrum of the polyethylene sample obtained by catalyst C. The overall distribution is unimodal, showing the characteristics of narrow overall molecular weight distribution, reflecting the characteristics of the corresponding single-active center catalyst for ethylene polymerization.

Table 3 lists polyethylene fractions determined by gel chromatography (GPC). Quantum and its distribution. In the table, M_w_ is the weight average molecular weight; M_n_ is the number average molecular weight; PDI = M_w_/M_n_ is the molecular weight distribution.

**Table 2..** Effect of Catalyst Structure on the Catalytic Activity and relative molecular mass

**Entry**	**Catalyst**	**Activity/(KgPE.mol^−1^Ti.h^−^**[^1]^)	**10^−6^M_v_**
1	C	78.56	1.48
2	C_1_	56.56	1.28
3	C_2_	1	0.08

**Table 3.** Polymer characterization results determined by GPC

**10^−6^M_W_**	**10^−^**[^6]^**M_n_**	**PDI**
1.60	0.65	2.46

**Figure 9.**
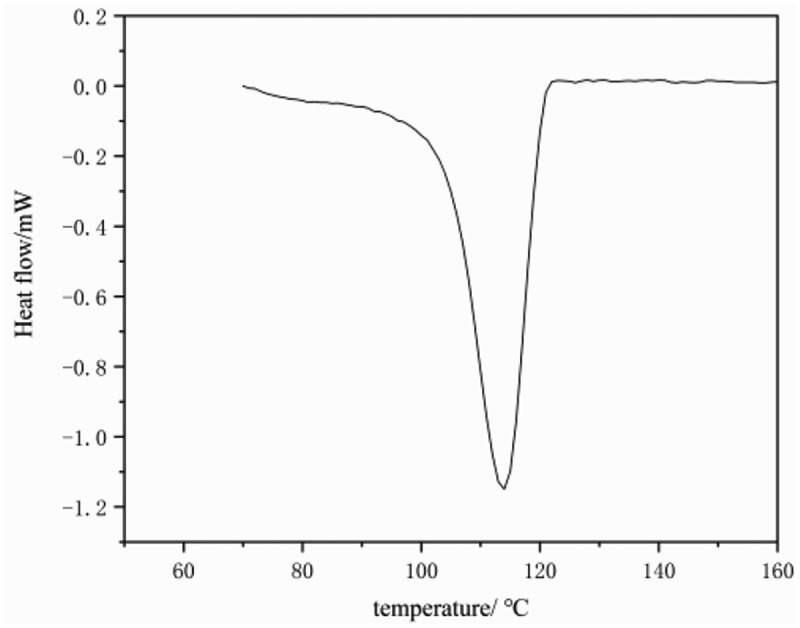
Figure 9．DSC crystal curves of PE (entry 4 in Table 1)

**Figure 8.**
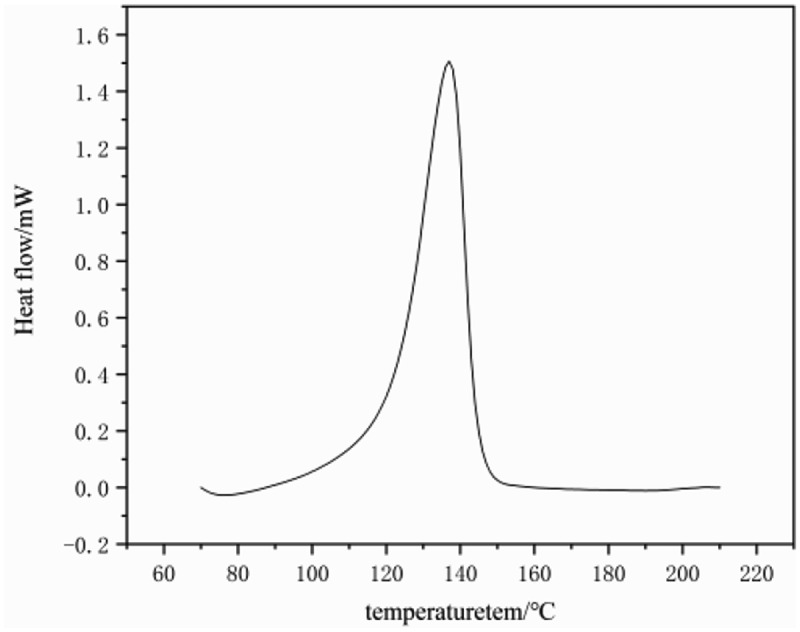
Viscoelastic properties of polyethylene (a) Storage and loss moduli and

Figure 8 and Figure 9 show the DSC curve of the polyethylene sample obtained from catalyst C. indicates that the melting peak of the obtained polyethylene is relatively narrow, thus supporting its high-temperature GPC data. The melting point of the sample reached 136 °C, Crystallization temperature 114°C, which is in line with the thermal performance characteristics of UHMW-PE [38].

### 3.8 Viscoelasticity of polyethylene

**Figure 10.**
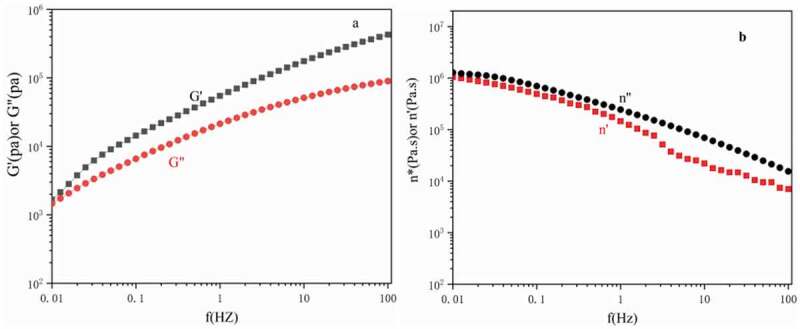
Viscoelastic properties of polyethylene (a) Storage and loss moduli and. (b) Complex viscosity and dynamic viscosity

At 220°C, Figure 10 show the dynamic storage and loss modulus (*G′* and *G″*), complex viscosity (*η″*), and dynamic viscosity (η′) of polyethylene as a function of frequency (*f*) are shown in Figures 9(a) and (b). In the entire frequency range of the study, the storage modulus was higher than the loss modulus, showing the high elasticity of polyethylene. The dynamic viscous flow properties of polyethylene are consistent with the characteristics of ultra-high molecular weight polyethylene [39]this is caused by the entanglement between macromolecular chains. The higher the molecular weight of the polymer, the easier it is for the polymer chains to entangle and interact. The high elasticity of polyethylene shows that polyethylene is an ultra-high molecular weight polyethylene. This result is consistent with the result of viscosity-average molecular weight determination.

## 3 Conclusions

In summary, the dendritic 3,5-di-tert-butylsalicylaldehyde ligand and its titanium metal catalyst were synthesized, and the structure was characterized by FTIR, 1 HNMR, UV-vis, ESI-MS, and ICP-MS. The actual structure is consistent with the theoretical design structure. This titanium metal catalyst was used as the catalyst precursor, under the activation of MAO, the catalyst system under different polymerization conditions (e.g., Al/Ti molar ratio, reaction time, reaction temperature, and polymerization pressure). The results show that the dendritic 3,5-di-tert-butylsalicylaldehyde titanium metal catalyst has good catalytic performance for ethylene polymerization. At the reaction temperature of 25°C, the reaction time was 30 min, and the ethylene pressure was 1.0. When the ratio of the amount of MPa and Al/Ti was 1,000, the catalytic activity can reach 78.56 kg PE/(mol Ti.h), which is much higher than that of monomolecular catalysts with similar structures and dendrimers with the low steric hindrance of steric substituents. The catalyst proves that the steric hindrance of the substituent has a significant effect on the dendritic titanium catalyst catalyzing ethylene. As the steric hindrance of the substituent increased, the catalytic activity and the molecular weight of the product also increased. In addition, the thermal properties and viscoelastic state of the catalyzed polyethylene samples were analyzed and characterized, and the results showed that the product was ultra-high-molecular-weight polyethylene.
